# Facilitating adherence to endocrine therapy in breast cancer: stability and predictive power of treatment expectations in a 2-year prospective study

**DOI:** 10.1007/s10549-017-4637-2

**Published:** 2018-01-12

**Authors:** Yiqi Pan, Sarah R. Heisig, Pia von Blanckenburg, Ute-Susann Albert, Peyman Hadji, Winfried Rief, Yvonne Nestoriuc

**Affiliations:** 1 Department of Psychosomatic Medicine and Psychotherapy, University Medical Center Hamburg-Eppendorf, Martinistraße 52, 20251 Hamburg, Germany; 2 Clinical Psychology and Psychotherapy, University of Hamburg, Von-Melle-Park 5, 20146 Hamburg, Germany; 3 Department of Clinical Psychology and Psychotherapy, Philipps-University of Marburg, Gutenbergstraße 18, 35032 Marburg, Germany; 4 AWMF-Institute for Medical Knowledge Management, Philipps-University of Marburg, Karl-von-Frisch-Str. 1, 35043 Marburg, Germany; 5 Department of Gynecology and Obstetrics, Krankenhaus Nordwest, Steinbacher Hohl 2-26, 60488 Frankfurt am Main, Germany; 6 Schön Klinik Hamburg Eilbek, University Clinic for Psychosomatic Medicine and Psychotherapy, Dehnhaide 120, 20081 Hamburg, Germany

**Keywords:** Adherence, Expectation, Endocrine therapy, Adverse events, Breast cancer

## Abstract

**Purpose:**

To identify modifiable factors predictive of long-term adherence to adjuvant endocrine therapy (AET).

**Methods:**

As part of a 2-year cohort study in primary care (*n* = 116), we investigated whether initial treatment expectations predict adherence at 24 months after controlling for demographic, medical, and psychosocial variables. Treatment expectations were measured as necessity–concern beliefs, expected side-effect severity, and expected coping with side effects. Their stability over time and differences of trajectories between the adherent and nonadherent group were examined.

**Results:**

Nonadherence at 24 months was 14.7% (*n* = 17). Side-effect severity at 3 months [OR 0.25, 95% CI (0.08, 0.81), *p* = 0.02] and necessity–concern beliefs [OR 2.03, 95% CI (1.11, 3.72), *p* = 0.02] were the sole predictors of adherence. Necessity–concern beliefs remained stable over 2 years, whereas expected side-effect severity (*p* = 0.01, *η*_p_^2^ = 0.07) and expected coping with side effects became less optimistic over time (*p* < 0.001, *η*_p_^2^ = 0.19), the latter particularly among nonadherers (*p* < 0.01, *η*_p_^2^ = 0.10).

**Conclusions:**

Patients’ initial necessity–concern beliefs about the AET and early severity of side effects affect long-term adherence. Expecting poor management of side effects may also facilitate nonadherence. We suggest that discussing benefits, addressing concerns of AET, and providing side-effect coping strategies could constitute a feasible and promising option to improve adherence in clinical practice.

**Electronic supplementary material:**

The online version of this article (10.1007/s10549-017-4637-2) contains supplementary material, which is available to authorized users.

## Introduction

Nonadherence rates in adjuvant endocrine therapy (AET) for breast cancer range from 41 to 72% at 5 years [36] including 30–47% patients who have discontinued [5, 25]. Nonadherence lead to a higher risk of all-cause mortality and recurrence compared to completion of treatment [16, 33]. Currently, three treatments are available: Tamoxifen, Aromatase Inhibitors, and Fulvestrant (used in the metastatic setting). With breast cancer being the most common cancer for women worldwide [27] and AET assigned for approximately 75% of all breast cancer cases [37], it is important to understand which factors are related to nonadherence. Meta-analyses and systematic reviews have reported that the most consistent demographic, clinical and treatment characteristics related to nonadherence in AET are younger age, follow-up care by a general practitioner versus an oncologist or gynecologist, increased costs, medication switch, higher frequency of hospitalizations, and treatment side effects [9, 34, 36].

Side effects include vasomotor symptoms, arthralgia, nausea, weight gain, vaginal dryness, and many others. Incidence rates of 21–43% were found for hot flushes and arthralgia in clinical trials [11, 15, 43] and indicate that side effects are common, can be burdensome, and for some, to such a degree that quality-of-life is largely affected [11]. However, as noted by Cahir et al. [9], it remains unknown whether it is the side effects per se or poor side-effect coping that leads to nonadherence. Aside from pharmacodynamics of the drug, side effects can be influenced by one’s expectations of side effects occurrence and intensity, a phenomenon known as the ‘nocebo-effect’ [14]. Studies with breast cancer patients undergoing chemotherapy or AET have found that expecting more side effects can lead to more actual side effects [12, 38, 41]. Given the relations between nonadherence and side effects, and side-effect and side-effect expectations, investigating the immediate relationship between side-effect expectations and adherence is warranted.

One expectation factor which has been frequently researched with regard to adherence is the patient’s perceived treatment necessity (e.g., to prevent recurrence) and related concerns (e.g., side effects, potential drug dependency). The underlying theoretical framework suggests that patients individually weigh their perceived necessity against their concerns and derive corresponding behavior [23]. Accordingly, if concerns outweigh necessity, nonadherent behavior would be more probable. The link between necessity–concern beliefs and adherence to AET has been found in a small body of studies [8, 18, 19, 28, 30, 42], including two longitudinal studies with a follow-up of 2 and 5 years [18, 30]. However, a recent review argues that the overall evidence remains tentative since most studies were cross-sectional [34] and some studies used unvalidated measures of necessity–concern beliefs [8, 18, 30, 42].

The goal of this study was to examine stability and predictive power of initial treatment expectations, i.e., expected side-effect severity, expected coping with side effects, and necessity–concern beliefs on adherence to AET after 24 months in a multivariate model along with clinical, demographic, and psychosocial characteristics. Also, up to now, it remains unknown whether treatment expectations change or remain stable over the course of the treatment. Furthering our understanding of modifiable factors like expectations could be helpful to design interventions with the objective to increase adherent behavior.

## Methods

### Patients and procedure

This sample was based off a clinical cohort study. Patients (*n* = 116) were women with hormone receptor-positive breast cancer or DCIS indicated for AET. Other adjuvant therapies were not an exclusion criterion. The recruitment process, further inclusion criteria, and study flow were described in detail elsewhere [38]. Informed consent was given by all patients before study inclusion. We standardized the amount of pre-knowledge about the AET as much as possible by providing all participants with prior validated information about beneficial effects and side effects of the treatment (verbally and using a leaflet) [22]. The ethics committee for medical research of the University of Marburg and the ethics committee of the medical chamber of Hamburg gave ethical approval.

### Measures

Except for necessity–concern beliefs and side effects, which were assessed at 3 months, all enlisted variables were collected around treatment start. For 21 patients, these variables were collected after they had started AET (*M* = 7 days, range 1–31 days). Treatment expectations and adherence were additionally assessed at 24 months. When patients had already discontinued AET at assessment time, they were instructed to refer to the time period shortly before discontinuation.

#### Demographic and medical characteristics

A semistructured interview and the clinical assessment in neuropsychiatry (SCAN) [51] were used to assess demographics, comorbidities, and concurrent medication. Disease information was collected from medical records. The generic assessment of side effects (GASE) [39] measured physical symptoms at treatment start (baseline symptoms) and side effects at 3-month follow-up. The questionnaire listed 45 symptoms including 36 of the most common adverse effects across different medications and 9 of which were specific of the AET [38]. A global question on severity and burden concluded the symptom listing (“Please indicate how much you have overall suffered from these symptoms in the last 7 days”). All symptoms including the global item were rated on a scale from 0 (*none*) to 3 (*severe*). We used the global scale due to its easier interpretation. In addition, we calculated the number of side effects and a mean severity score by averaging the severity of 44 symptoms (menstrual symptoms excluded since 80% of our sample was postmenopausal).

#### Psychosocial characteristics

The hospital anxiety and depression scale (HADS) [53] measured anxiety and depression, calculated as the sum of all items [6]. Quality-of-life was assessed by the global quality-of-life scale of the questionnaire of the European organization for research and treatment of cancer (EORTC QLQ-C30) (“how would you rate your overall quality of life during the last week?”) [1].

#### Treatment expectations

The beliefs about medicines questionnaire (BMQ) [24] assessed necessity–concern beliefs about the AET. Cronbach’s Alpha ranged from *α* = 0.76 to *α* = 0.77 (necessity subscale) and from *α* = 0.66 to *α* = 0.74 (concern subscale). Necessity–concern beliefs had a range from − 4 to 4 and were obtained by subtracting “concern” from “necessity.” Expected side-effect severity and expected coping with side effects were assessed using the GASE-Expect [38, 39]. Patients indicated expected severity and burden of future side effects (0 = *none expected* to 3 = *severe*) and how well they would be able to cope with them (0 = *poorly* to 3 = *well*).

#### Adherence

Adherence was assessed via self-report with a validated single item (“how many pills have you actually taken during the last week?”) [54]. Patients who discontinued treatment were additionally requested to specify the reason (open question). We used an 80% criterion to categorize adherers and nonadherers. This criterion is commonly applied for adherence to AET [4, 50] and has proven critical with respect to mortality reduction [32]. Due to our overall small sample size, we did not conduct analyses for adherence and persistence separately.

### Data analyses

Missing values analysis was imputed using multiple imputation algorithms [40]. Entire missing questionnaires were not imputed.

We included *n* = 116 patients in the adherence analyses. A hierarchical logistic regression was conducted with demographic and medical variables, baseline symptom severity, and side-effect severity, being entered in the first step. Anxiety and depression and quality-of-life were entered in the second step, followed by necessity–concern beliefs, expected side-effect severity, and expected coping with side effects in the third step. Three patients dropped out of the study at 3 months and had already discontinued treatment at that time. Since the majority of patients do not resume therapy after discontinuation [5], we included these patients as nonadherers. However, the possibility that treatment was resumed could not be excluded. Hence, we conducted a sensitivity analysis under exclusion of these three patients. Further sensitivity analyses were performed by substituting the global side-effect severity scale by the mean severity score, and numbers of side effects in the regression analysis, respectively.

Stability analysis included further data at 24 months and was conducted with *n* = 102 participants. A 2 × 2 mixed analyses of variance (ANOVAs) was conducted with adherence status at 24 months as the between-subjects factor (adherent vs. nonadherent) and time as the within-subject factor (treatment start vs. 24-month follow-up).

## Results

### Patient characteristics

Table 1 shows the sample characteristics at treatment start. The mean age was *M* = 55.40 years (SD = 9.97). Most women had a partner (74%), reported primary or secondary education (82%), were diagnosed with breast cancer stage I (52%) and had at least one medical comorbidity (70%). The average number of prescription medications besides AET was *M* = 1.5 (SD = 1.50). After 3 months of AET, 84% of patients reported side effects of mild (43%) or moderate (36%) intensity. Quality-of-life was rated as good (*M* = 4.91, SD = 1.41) and anxiety and depression as rather low (*M* = 8.24, SD = 6.13). Necessity–concern beliefs were close to zero, indicating that on average, patients reported concerns equaled the perceived necessity of the treatment (*M* = 0.38, SD = 1.11). Expected side-effect severity (*M* = 1.21, SD = 0.61) was rated as mild and expected coping with side effects (*M* = 1.91, SD = 0.60) was rated as rather well. The adherence rate was 85% at 24 months. Of the 17 patients (15%) who were classified as nonadherers, four women discontinued AET within the first three months, whereas 11 patients discontinued between months 3 and 24. Two had taken less than 80% of the pills at 24-month follow-up. The exact time of discontinuation was known for *n* = 7 patients. On average, these patients discontinued after 17 months (range 10–24 months).

**Table 1:** Patients’ sociodemographic and clinical characteristics (*N* = 116)

Characteristic	*n* (%) or mean (SD), range
Age, years	55.40 (9.97, 26–79)
Married/partner	86 (74.1%)
< 13 years of education	95 (81.9%)
Tumor staging UICC	
0	4 (3.4%)
I	60 (51.7%)
II	36 (31.0%)
III	12 (10.3%)
IV	4 (3.4%)
Comorbid health condition	
None	35 (30.2%)
At least one	81 (69.8%)
Number of concurrent medications	
0	39 (33.6%)
1	29 (25.0%)
2	20 (17.2%)
3	16 (13.8%)
4	4 (3.4%)
5 or more	8 (6.9%)
Baseline symptom severity	
No symptoms	19 (16.4%)
Mild symptoms	71 (61.2%)
Moderate symptoms	26 (22.4%)
Type of AET medication	
Tamoxifen	50 (43.1%)
Aromatase Inhibitor	66 (56.9%)
Medication switch within the first 3 months	7 (6.0%)
Side-effect severity at 3 months	
No side effects	18 (15.5%)
Mild side effects	50 (43.1%)
Moderate side effects	42 (36.2%)
Severe side effects	6 (5.2%)
Quality-of-life (scale range: 0–7)	4.91 (1.41)
Anxiety and depression (scale range: 0–21)	8.24 (6.13)
Necessity–concern beliefs (scale range: − 4–4)^a^	0.38 (1.11)
Expected side-effect severity (scale range: 0–3)	1.21 (0.61)
Expected coping with side effects (scale range: 0–3)	1.91 (0.60)
Adherence at 24 months	
Adherent (≥ 80% intake)	99 (85.3%)
Nonadherent (< 80% or discontinuation)	17 (14.7%)
Discontinuation at 3 months	4 (3.4%)
Discontinuation between 3 and 24 months	11 (9.5%)
< 80% at 24-month FU	2 (1.7%)

*UICC* union for international cancer control, *AET* adjuvant endocrine therapy, *FU* follow-up

^a^Positive scores indicate the perceived necessity to outweigh concerns

#### Prediction of adherence

Variables bivariately associated with adherence were lower side-effect severity at 3 months (*r* = − 0.33, *p* < 0.001), lower anxiety and depression (*r* = − 0.18, *p* = 0.049), higher necessity–concern beliefs (*r* = 0.28, *p* = 0.002), and lower expected side-effect severity (*r* = − 0.22, *p* = 0.02) (supplement 1).

In the regression model, adherence was predicted by side-effect severity at 3 months [OR 0.25, 95% CI (0.08, 0.81), *p* = 0.02], and necessity–concern beliefs [OR 2.03, 95% CI (1.11, 3.72), *p* = 0.02] (Table 2). The model explained 36% of the variance in adherence (Nagelkerke’s *R*^*2*^) and obtained significance (*χ*^2^ (15) = 26.27, *p* = 0.04). Adherence status was predicted correctly in 87.9% of the cases. Sensitivity analyses without the three patients who were nonadherent at 3 months and lost to follow-up subsequently showed the same results.

**Table 2:** Multiple logistic regression model for predictors of 24-month adherence to AET

Predictors	Model 1		Model 2		Model 3	
	OR [95% CI]	*p*	OR [95% CI]	*p*	OR [95% CI]	*p*
Age	1.03 [0.95–1.11]	0.45	1.02 [0.94–1.11]	0.61	1.04 [0.95–1.14]	0.38
Marital status						
Single = 0						
Married/partner = 1	0.89 [0.24–3.31]	0.86	0.98 [0.25–3.86]	0.98	0.75 [0.17–3.36]	0.70
Education						
≤ 13 years = 0						
> 13 years = 1	1.46 [0.29–7.46]	0.65	1.60 [0.30–8.63]	0.59	1.95 [0.31–12.41]	0.48
Staging	1.28 [0.63–2.62]	0.50	1.32 [0.64–2.73]	0.45	1.17 [0.53–2.54]	0.70
Physical comorbidity						
None = 0						
At least one = 1	1.00 [0.15–6.83]	0.99	1.03 [0.15–7.22]	0.98	0.84 [0.11–6.62]	0.87
Number of concurrent medications	0.73 [0.44–1.20]	0.21	0.75 [0.45–1.25]	0.26	0.59 [0.33–1.08]	0.09
Baseline symptom severity	1.39 [0.44–4.36]	0.58	1.50 [0.45–4.99]	0.51	1.89 [0.45–7.87]	0.38
Type of AET						
Tamoxifen = 0						
Aromatase inhibitor = 1	2.63 [0.67–10.30]	0.17	2.58 [0.66–10.18]	0.18	2.40 [0.55–10.80]	0.24
Medication switch						
No switch = 0						
Switch within first 3 M = 1	0.29 [0.01–6.52]	0.44	0.40 [0.02–9.27]	0.57	0.49 [0.004–13.51]	0.49
Side-effect severity at 3 M	**0.22** [0.08–0.62]	0.004**	**0.22** [0.08–0.64]	0.005**	**0.25** [0.08–0.81]	0.02*
Quality-of-life			0.89 [0.52–1.52]	0.68	0.84 [0.47–1.51]	0.56
Anxiety and depression			0.95 [0.85–1.08]	0.43	0.95 [0.83–1.08]	0.41
Necessity–concern beliefs					**2.03** [1.11–3.72]	0.02*
Expected side-effect severity					0.72 [0.19–2.82]	0.64
Expected coping with side effects					0.94 [0.23–3.75]	0.93
Model fit indices and significant tests						
Nagelkerke’s *∆R*^2^	0.26		0.01		0.09	
	*χ*^2^ (10) = 18.22, *p* = 0.05		*χ*^2^ (2) = 0.64, *p* = 0.73		*χ*^2^ (3) = 7.40, *p* = 0.06	
Total Nagelkerke’s *R*^2^			**0.27**		**0.36**	
			*χ*^2^ (12) = 18.87, *p* = 0.09		*χ*^2^ (15) = 26.27, *p* = 0.04*	

*Note N* = 116. Tests which obtained significance are in boldface

*OR* odds ratio, *CI* confidence interval, *AET* adjuvant endocrine therapy, *M* = months

* *p* < 0.05, ** *p* < 0.01

We repeated the analysis and substituted the global items of baseline symptom severity and side-effect severity by the respective mean intensity score of the 44 listed symptoms. We obtained the same result pattern with significant multivariate associations between adherence and side-effect severity [OR 0.53, 95% CI (0.003, 0.91), *p* = 0.04], and necessity–concern beliefs [OR 2.42, 95% CI (1.19, 4.92), *p* = 0.02; Model: Nagelkerke’s *R*^2^ = 41%*; χ*^2^ (15) = 30.66, *p* = 0.01]. Interestingly, when number of side effects (controlled for number of baseline symptoms) was entered instead of side-effect severity, its link to adherence was not present anymore [OR 0.90, 95% CI (0.79, 1.02), *p* = 0.09]. Necessity–concern beliefs, however, remained predictive of adherence [OR 2.14, 95% CI (1.13, 4.06), *p* = 0.02].

### Stability of treatment expectations

We then compared treatment expectations at treatment start and at 24-month follow-up. Table 3 shows means, standard deviations and correlations by adherence status of both time points.

**Table 3:** Treatment expectations at treatment start and at 24-month follow-up (*M* [SD])

	Total sample			Adherent			Nonadherent		
	Treatment start (*n* = 116)	24 months (*n* = 102)	Correlation	Treatment start (*n* = 99)	24 months (*n* = 96)	Correlation	Treatment start (*n* = 17)	24 months (*n* = 8)^a^	Correlation
Necessity–concern beliefs	0.38 (1.11)	0.33 (1.03)	0.52**	0.51 (0.88)	0.44 (0.94)	0.38**	-0.38 (1.84)	-1.07 (1.22)	0.85*
Expected side-effect severity	1.21 (0.61)	1.39 (0.78)	0.24*	1.15 (0.58)	1.31 (0.75)	0.16	1.53 (0.72)	2.13 (0.84)	0.57
Expected coping with side effects	1.91 (0.60)	1.66 (0.77)	0.34**	1.94 (0.53)	1.74 (0.70)	0.36**	1.71 (0.92)	0.63 (0.74)	0.27

The necessity–concern beliefs scale ranges from − 4 to 4. Higher values indicate the perceived necessity to outweigh concerns. The scales for expected side-effect severity and expected coping with side effects range from 0 to 3. Significant correlations are in boldface. Data at 24 months was available for *n* = 102 patients due to lost to follow-up

*M* mean, *SD* standard deviation, *Start* treatment start, *FU* 24-month follow-up

*p < 0.05, **p < 0.01

^a^Of *n* = 8 nonadherent patients who specified treatment expectations, 6 discontinued treatment, 2 took less than 80% of the pills

**Fig. 1:**
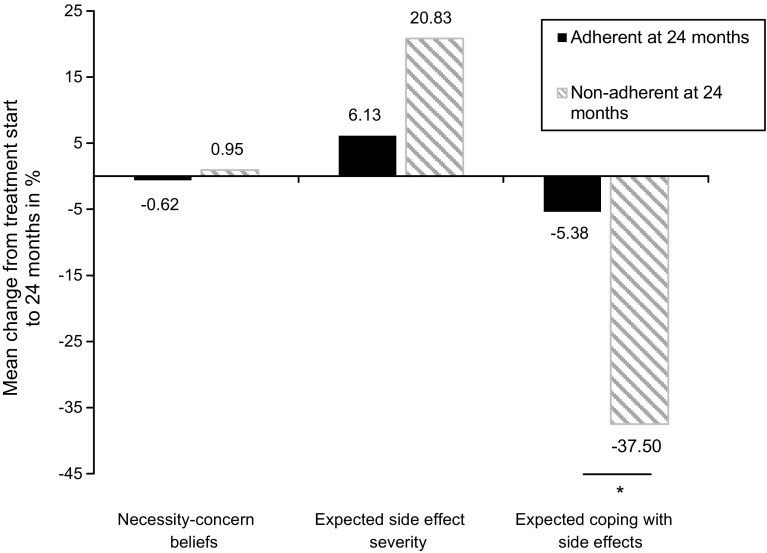
Change of patients’ treatment expectations over 2 years as a function of 24 months adherence status to adjuvant endocrine therapy. Percentages are relative to the respective scale range. A significant Time × Adherence interaction effect was found for expected coping with side effects (*p* < 0.01)

Results of the mixed ANOVA showed no main effect of time for necessity–concern beliefs [*F* (1, 100) = 0.004, *p* = 0.95, *η*_*p*_^2^ < 0.001], indicating that it did not change over time. However, side effects were expected to become more severe [*F* (1, 98) = 6.78, *p* = 0.01, *η*_*p*_^2^ = 0.07], and expected coping with side effects was rated as more poorly over the course of 2 years [*F* (1, 99) = 22.57, *p* < 0.001, *η*_*p*_^2^ = 0.19]. Significant between-subject effects indicate that adherent and nonadherent patients overall differed in their treatment expectations [*F*_*Necessity*-*concern beliefs*_ (1, 100) = 24.77, *p* < 0.001, *η*_*p*_^2^ = 0.20; *F*_*expected side effect severity*_ (1, 98) = 9.40, *p* = 0.003, *η*_*p*_^2^ = 0.09; *F*_*expected coping with side effects*_ (1, 99) = 11.42, *p* = 0.001, *η*_*p*_^2^ = 0.10]. A significant interaction effect for Adherence × Time was found for expected coping with side effects [*F* (1, 99) = 11.42, *p* < 0.01, *η*_*p*_^2^ = 0.10], yet neither for necessity–concern beliefs [*F* (1, 100) = 0.10, *p* = 0.75, *η*_*p*_^2^ = 0.001], nor for expected side-effect severity [*F* (1, 98) = 2.00, *p* = 0.16, *η*_*p*_^2^ = 0.02]. We computed the change over time of each expectation variable relative to their scale range. As shown in Fig. 1, necessity–concern beliefs remained stable, whereas expected coping with side effects became less optimistic by 5.4 and 37.5% among adherent and nonadherent patients. Expected side-effect severity trajectories increased by 6.1 and 20.83%. However, this difference in trajectory was not significant. Additional analysis on the stability of side-effect severity showed no significant effects, neither for Time [*F* (1, 100) = 0.48, *p* = 0.49, *η*_*p*_^*2*^ = 0.01], nor for the Adherence × Time interaction [*F* (1, 100) = 0.58, *p* = 0.45, *η*_*p*_^*2*^ = 0.01].

### Reasons for discontinuation

**Fig. 2:**
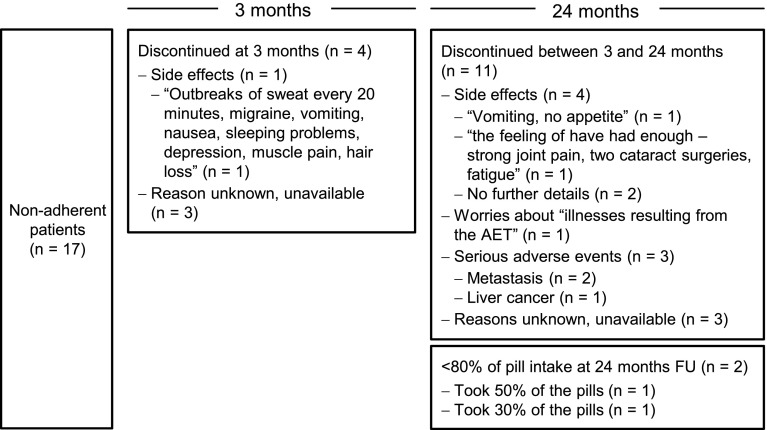
Patient-reported reasons for discontinuing adjuvant endocrine treatment

The reasons for discontinuation are depicted in Fig. 2. Five patients indicated to have discontinued due to side effects. One and three patients indicated to have discontinued due to worries about potential serious adverse side effects of the AET and actual serious adverse events (metastasis; liver cancer), respectively. Another 6 patients reported to have discontinued yet were unattainable for further inquiries.

## Discussion

In a prospective cohort study in primary care with 116 patients, we documented a nonadherence rate of 14.7% after 2 years. Initial side-effect severity and necessity–concern beliefs predicted long-term adherence over and above the influence of sociodemographic, other medical, and psychosocial characteristics. Expected side-effect severity and expected coping with side effects did not predict adherence. Necessity–concern beliefs remained stable on an overall medium level over the course of 2 years, whereas side-effect and coping expectations became less optimistic over time. The trajectories of coping expectations differed by adherence status at 24 months with expected coping being less optimistic among the nonadherent than the adherent patients.

Our findings indicate that, given a scale range from − 4 to 4, a patient with a necessity–concern beliefs value of 1 (i.e., necessity slighter higher than concerns) is two times more likely to be adherent than a patient with necessity–concern beliefs of 0 (i.e., necessity and concerns equally high). A patient with necessity–concern beliefs of 2, again, is four times more likely to be adherent. As for side effects, a patient with mild side effects is four times more likely to be adherent than a patient with moderate side effects. Our finding adds to the line of studies which linked necessity–concern beliefs to AET adherence [2, 8, 18, 19, 28, 30, 42, 46, 52]. Hereby, our study is the first to report this relationship in a prospective design over 24 months using a validated questionnaire of necessity–concern beliefs. These results also indicate that side-effect severity and necessity–concern beliefs play a pivotal role in AET adherence over and above demographic and clinical characteristics, which did not contribute to the prediction. Except for younger age, the latter finding aligns with reviews and meta-analyses which reported inconsistent associations between adherence and most demographic and clinical characteristics [9, 34, 36], including quality-of-life [45], depression [31], and medication switch, which facilitated adherence in some studies, and nonadherence in others [34].

The influence of side effects on adherence was also implied by patients’ self-reports, given that five out of 15 patients named “side effects” as their reason for discontinuation. Notably, whereas side-effect severity predicted adherence in the logistic regression model, numbers of side effects did not, suggesting that the link may differ depending on how side effects are operationalized. This has also been reflected by reviews [9, 34], which reported the relationship between side effects and adherence to be inconsistent across studies. We propose that the link is unequivocal since it might be influenced by side-effect appraisal. For example, a recent qualitative study found that women were more willing to accept side effects when they were aware that the treatment was necessary to prevent recurrence [35]. Moreover, our findings indicated that side-effect management may play a role considering adherence, giving that, especially among the nonadherent group, expected coping with side effects became less optimistic over time (37.5% vs. 5.4% for nonadherent and adherent patients). As side-effect severity did not change significantly from 3 to 24 months—neither in the overall sample nor in the nonadherent or adherent patient group—these results suggest that expected low self-efficacy to cope with future side effects may influence adherence behavior which goes beyond side effects per se. Taken together, we suggest that burden of side effects may affect adherence behavior, yet should be examined in the context of further related factors, e.g., necessity–concern beliefs and side-effect management.

In the literature, side-effect management is acknowledged as important to maintain or increase adherence to AET [21, 48], and a variety of recommendations are suggested for different side-effect categories [17]. However, systematic research examining the degree of its implementation is lacking, whereas trials which investigate the efficacy of those strategies are few (e.g. for hot flushes, see [44] for arthralgia, see [3, 13]). Although due to the correlative nature of the data, we cannot answer the question whether patients discontinue treatment because they expect their management to be ineffective or whether they discontinue first and rationalize their behavior thereafter, our findings nonetheless reinforce the importance of side-effect coping in AET. Its pivotal role considering adherence optimization has also been pointed out by both patients [46, 48] and practitioners [49] in prior qualitative studies. Furthermore, in a trial of cognitive-behavioral therapy for vasomotor symptoms of breast cancer survivors, beliefs about coping and control of hot flushes were found to be the main mediator of how burdensome symptoms were perceived after the therapy [10]. Overall, more studies are needed to investigate whether poor coping abilities lead to nonadherence, and to which degree patients wish for further support.

Limitations of this study include a selection bias. The enhanced information about AET which was provided as part of the study might have appealed to women who were more open to the treatment or perhaps even increased patients’ willingness to be adherent. We believe the latter bias to be minor since interventions which aimed at improving adherence by providing information were found to be not effective [26]. Nonetheless, our nonadherence rate was lower than the rates found in large health plan studies (15% vs. 27%–45%) [20, 29], which indicates a bias which might have weakened the external validity of this study. By means of a subjective measure to assess adherence, we obviously cannot exclude a reporting bias. However, a single-item, self-report adherence measure has been found to be associated with estrogen serum levels [7]. Also, we assessed necessity–concern beliefs at 3 months, whereas side-effect and coping expectations were assessed at treatment start. Since expectations might be affected by treatment experiences, comparability of these factors’ trajectories may be limited. Finally, the numbers of nonadherers who were included in stability analyses (*n* = 8) and the numbers of patients who have specified the reason for discontinuation were very small (*n* = 9). Thus, interpretations must be viewed in light of limited representativeness.

## Conclusions

In the context of the present body of research, our findings show a coherent picture indicating the importance of patients’ understanding of the individual necessity of AET. More specifically, it seems important that necessity beliefs outweigh individual concerns, which are an inherent part of patients’ treatment evaluations. Overall, patients reporting more necessity beliefs than concerns and experiencing fewer initial side effects were more likely to be adherent in the long term. Also, positive coping expectations with side effects of the AET decreased over time in nonadherent compared with adherent patients.

To reduce the perceived burden of side effects, practitioners could support patients’ side-effect management [47]. Follow-up visitations could be used to screen patients with poor coping expectations, who are then provided with individual management strategies. In summary, by addressing benefits of the AET, by exploring potential concerns of a patient, and by offering coping strategies during the course of the treatment, adherence might be optimized in the long term.

## Electronic supplementary material

Below is the link to the electronic supplementary material.

Supplementary material 1 (DOC 55 kb)
