# Supplementary material for: Facilitating adherence to endocrine therapy in breast cancer: stability and predictive power of treatment expectations in a 2-year prospective study
Source: Breast Cancer Res Treat. 2018 Jan 12;168(3):667–77. doi: 10.1007/s10549-017-4637-2 (PMC5842254; doi:10.1007/s10549-017-4637-2)
Supplement: Supplementary file 1 — Supplementary material 1 (DOC 55 kb) [file 10549_2017_4637_MOESM1_ESM.doc]

Pan Y, Heisig S, von Blanckenburg P, Albert U-S, Hadji P, Rief W, Nestoriuc Y. **Facilitating adherence to endocrine therapy in breast cancer: Stability and predictive power of treatment expectations in a 2-year prospective study**

Breast Cancer Research and Treatment (submitted)

**Corresponding author:** Yiqi Pan, y.pan@uke.de

University Medical Center Hamburg-Eppendorf, Department of Psychosomatic Medicine and Psychotherapy, Hamburg, Germany

**Supplement 1**

Intercorrelations of variables included in the adherence analysis

|  | Variables | 1 | 2 | 3 | 4 | 5 | 6 | 7 | 8 | 9 | 10 | 11 | 12 | 13 | 14 | 15 |
| --- | --- | --- | --- | --- | --- | --- | --- | --- | --- | --- | --- | --- | --- | --- | --- | --- |
| 1 | Age | 1 |  |  |  |  |  |  |  |  |  |  |  |  |  |  |
| 2 | Marital status^a^ | **-.23*** | 1 |  |  |  |  |  |  |  |  |  |  |  |  |  |
| 3 | Education^b^ | -.17 | .02 | 1 |  |  |  |  |  |  |  |  |  |  |  |  |
| 4 | Staging | .02 | .01 | -.03 | 1 |  |  |  |  |  |  |  |  |  |  |  |
| 5 | Physical comorbidity^c^ | **.30**** | -.17 | -.08 | .03 | 1 |  |  |  |  |  |  |  |  |  |  |
| 6 | Number of concurrent medications | **.49**** | -.16 | -.14 | .09 | **.58**** | 1 |  |  |  |  |  |  |  |  |  |
| 7 | Baseline symptom severity | -.13 | -.10 | -.01 | .08 | .003 | .01 | 1 |  |  |  |  |  |  |  |  |
| 8 | Type of AET^c^ | **.36**** | -.08 | -.13 | **.32**** | .11 | **.21*** | -.08 | 1 |  |  |  |  |  |  |  |
| 9 | Medication switch^d^ | .01 | -.10 | .07 | -.004 | -.07 | .01 | .09 | -.07 | 1 |  |  |  |  |  |  |
| 10 | SE severity at 3M | -.01 | **-.19*** | -.04 | .04 | **.35**** | .09 | **.40**** | -.08 | .08 | 1 |  |  |  |  |  |
| 11 | Quality of life | -.01 | **.20*** | .18 | -.18 | **-.23*** | -.11 | **-.36**** | -.10 | -.04 | **-.35**** | 1 |  |  |  |  |
| 12 | Anxiety and depression | -.12 | -.15 | **-.19*** | -.15 | .16 | .08 | **.36**** | .09 | -.05 | **.34**** | **-.49**** | 1 |  |  |  |
| 13 | Necessity-concern beliefs | .07 | -.02 | .11 | .16 | -.02 | .13 | -.16 | .08 | -.08 | **-.28**** | **.24**** | -.15 | 1 |  |  |
| 14 | Expected side effect severity | **-.25**** | **-.19*** | -.01 | -.07 | .007 | -.15 | **-33**** | .01 | .09 | **.30**** | **-.20*** | **-23*** | **-.26**** | 1 |  |
| 15 | Expected coping with side effects | .11 | .10 | <.001 | .08 | -.19 | .15 | **-.29**** | .01 | **-.26**** | **-.35**** | **.34**** | **-.34**** | **.28**** | **-.44**** | 1 |
| 16 | Adherence | -.02 | .09 | .01 | -.001 | -.17 | -.14 | -.12 | -.12 | .003 | **-.33**** | .11 | **-.18*** | **.28**** | **-.22*** | .14 |

*Note.* *N* = 116. AET = Adjuvant endocrine therapy; M = month. All predictors were assessed at treatment start, except for side effects and necessity-concern beliefs, which were assessed at 3 months. Significant correlations are in boldface.

^a^ Marital status: 0 = single; 1 = married/partner.

^b^ Education: 0 = ≤ 13 years; 1 = >13 years.

^c^ Co-morbid health condition: 0 = none; 1 = at least one

^c^ Type of AET medication: 0 = tamoxifen; 1 = aromatase inhibitor

^d^ Medication switch: 0 = no switch; 1 = switch within the first 3 months of intake

**p* < .05, ***p* < .01.
